# Pediatric Lymphangioma of the Tongue, From Diagnosis to Surgery: A Case Report

**DOI:** 10.1155/crid/8570180

**Published:** 2026-05-03

**Authors:** Federica Pulicari, Matteo Pellegrini, Sabrina Darwish, Martina Bosisio, Anita Groppi, Eleonora Segna, Francesca De Martino, Elisabetta Khun, Andrea Scribante, Francesco Spadari

**Affiliations:** ^1^ Maxillofacial Surgery and Dental Unit, Foundation IRCCS Cà Granda Ospedale Maggiore Policlinico, Milan, Italy; ^2^ Department of Biomedical, Surgical and Dental Sciences, University of Milan, Milan, Italy, unimi.it; ^3^ Section of Dentistry, Department of Clinical, Surgical, Diagnostic and Pediatric Sciences, University of Pavia, Pavia, Italy, unipv.eu; ^4^ Pathology Unit, Foundation IRCCS Ca′ Granda Ospedale Maggiore Policlinico, Milan, Italy

**Keywords:** cryotherapy, dentistry, laser therapy, lymphatic malformation, maxillofacial surgery, oral surgery, tongue

## Abstract

**Introduction:**

Lymphangioma is a rare pediatric benign lesion caused by a congenital malformation of the lymphatic system. Most often (50%–70%) lymphatic malformations affect skin and subcutaneous tissue of the head and neck region, rarely the oral cavity. Intraoral lymphatic malformation occurs most commonly on the dorsum of the tongue, followed by the palate, buccal mucosa, gums, and lips. Lymphatic malformations are clinically and radiologically classified into three types, according to the cystic size: microcystic (< 1 cm), macrocystic (> 1 cm), and mixed. Differential diagnoses for lingual lymphatic malformation include other vascular malformations, neurofibromas, thyroglossal cysts, and congenital hypothyroidism. Treatment is usually multidisciplinary. Early diagnosis is essential to prevent functional complications such as difficulties in speech, mastication, swallowing, and, in severe cases, airway obstruction. Imaging techniques including ultrasound, MRI, and CT are fundamental to assessing the lesion′s extension and relationship with surrounding structures. Histopathological confirmation remains the gold standard for definitive diagnosis. Therapeutic approaches include surgical excision, sclerotherapy, laser therapy, and in selected cases, systemic pharmacological treatments. Complete surgical removal may be challenging due to the infiltrative nature of the lesion and proximity to vital anatomical structures. Recurrence is relatively common, especially in microcystic and mixed types. Prognosis is generally favorable when appropriate management is achieved, although long‐term follow‐up is required.

**Methods:**

A 4‐year‐old female patient presented for a clinical consultation visit due to a lesion on the dorsum of the tongue. The local objective clinical examination revealed a lesion of the dorsum of the tongue, raised, erythematous, and nonbleeding. The size was about 10–12 mm and was not painful on palpation. Topical miconazole therapy was recommended at first, suspecting a fungal superinfection. Head and neck MRI with and without contrast was prescribed for suspected vascular lesions, but the result was not conclusive. In the end, surgical excision was programmed, and histological analysis confirmed the lymphatic malformation.

**Results:**

This case shows the efficacy of surgical treatment in tongue lymphatic malformation. In fact, the surgical treatment successfully confirmed the nature of the lesion, alleviating patient discomfort and improving the patient′s quality of life. Postoperative healing was uneventful at 1‐month follow‐up.

**Conclusions:**

Recognition of lymphatic malformations in rare locations such as the oral cavity is essential to prevent misdiagnosis and ensure timely, appropriate management.

## 1. Introduction

Lymphatic malformations (historically termed lymphangiomas) are rare, benign vascular anomalies of the lymphatic system, most commonly presenting in pediatric populations due to developmental errors during embryogenesis, frequently driven by somatic mosaic mutations in genes such as PIK3CA and related pathways [1, 2]. The International Society for the Study of Vascular Anomalies (ISSVA) classifies these lesions as low‐flow vascular malformations, with approximately 50%–70% occurring in the head and neck region, and a minority involving the oral cavity, particularly the tongue (about 6% of cases) [3–5]. Most lymphatic malformations are diagnosed in infancy or early childhood, with nearly half visible at birth and up to 90% manifesting by age two, although rare adult‐onset cases have been reported [6].

Clinically, lymphatic malformations are stratified into macrocystic, microcystic, and mixed subtypes based on imaging and histopathology, with microcystic lesions predominating in the tongue and oral mucosa [3–5]. These lesions typically present as painless, slow‐growing masses, with growth proportional to the patient′s development; acute enlargement may occur secondary to infection, inflammation, or hemorrhage [3]. Etiology is primarily genetic, but environmental factors such as maternal viral infections and substance abuse have been implicated [2].

Differentiation from hemangiomas is essential: hemangiomas are vascular tumors with GLUT1 positivity, whereas lymphatic malformations lack this marker and are closely associated with the overlying epithelium [3]. Diagnosis relies on clinical examination and imaging, with magnetic resonance imaging (MRI) preferred for delineating extent and involvement of adjacent structures [3, 7]. Management is individualized, with options including observation, sclerotherapy, surgery, and targeted medical therapies such as sirolimus, depending on lesion type, location, and symptoms [1–8]. Infants and young children are at the highest risk for developing tongue lymphatic malformations, with most cases diagnosed before age two and nearly half present at birth [9, 10]. The risk is further elevated in individuals with genetic syndromes such as Turner syndrome, Noonan syndrome, trisomy, PIK3CA‐related overgrowth syndromes (PROS), and other congenital conditions associated with somatic mosaic mutations in the PI3K/AKT/mTOR (mechanistic target of rapamycin) pathway [11, 12]. These mutations drive abnormal lymphatic endothelial cell proliferation and malformation development.

Patients with microcystic or mixed‐type malformations involving the tongue and floor of mouth, especially those with extensive or bilateral disease (stage V by de Serres classification), are most likely to experience complications. These complications include airway compromise, dysphagia, speech impairment, and significant morbidity, with lower functional scores and poorer outcomes compared with macrocystic or localized lesions [2, 3]. Due to the anatomical location of the tongue within the oral cavity and its proximity to the upper airway, lymphatic malformations in this site may pose a risk of airway compromise, particularly in infants and young children. Progressive enlargement, inflammation, or hemorrhage can lead to obstruction, feeding difficulties, and respiratory distress, making early recognition and appropriate management essential [10–14]. Children with underlying syndromic associations or immunodeficiency may also be at increased risk for infection, hemorrhage, and hospitalization [10–14].

We chose to document this case report to highlight and critically assess the diagnostic and therapeutic pathway for lingual lymphatic malformation in the pediatric population, with the aim of contributing to the understanding of optimal clinical management for this rare condition. To enhance transparency and completeness, the CARE checklist is provided with supporting information. Adherence to CARE guidelines has been shown to improve the quality of case report reporting in high‐impact journals. [15, 16]. Including the checklist facilitates reproducibility and critical appraisal by readers and reviewers [17] This approach aligns with current recommendations for standardized reporting in clinical research [17].

## 2. Case Report

### 2.1. Patient Information

**Figure 1:**
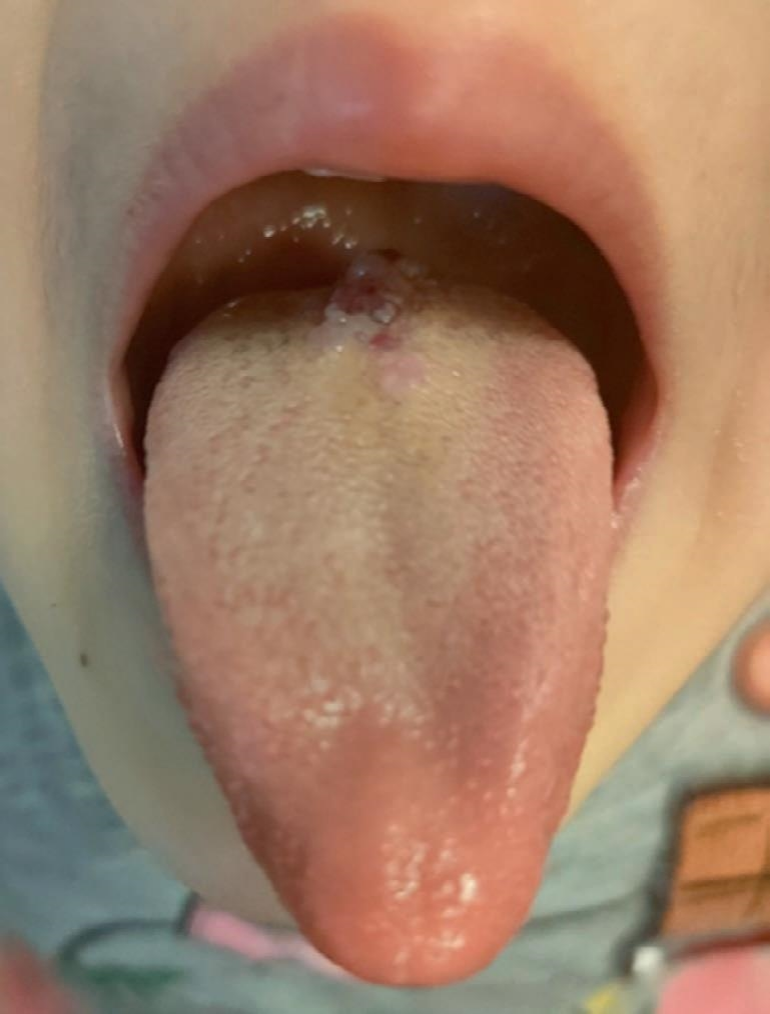
Initial clinical appearance of the lesion on the tongue.

A 4‐year‐old female patient presented to observation at the Oral Medicine and Pathology Unit of Fondazione IRCCS Cà Granda Ospedale Maggiore Policlinico in Milan, in December 2022 for a lesion on the dorsum of the tongue. Anamnesis stated the absence of allergies and no other pathological conditions, personal or familial, associated. The local objective clinical examination revealed a median lesion of the dorsum of the tongue, esophitic, erythematous, and nonbleeding, which the parents report to be present from April 2022 (Figure 1). The lesion measured approximately 10–12 mm in its greatest dimension and was not painful on palpation. The patient did not report any reduction in tongue movement. The lesion was soft in consistency, nonpulsatile, without bruit on auscultation, and did not exhibit blanching on digital pressure.

Multiple caries of the deciduous teeth and abscesses at the level of the left upper arch with gingival fistula in correspondence with Element 6.4 were observed.

Considering the presentation and the localization, the lesion observed was compatible with median rhomboid glossitis. Topical miconazole therapy was recommended, and a follow‐up visit was scheduled in 10 days. The patient followed the therapy prescribed. To the local clinical examination, the mucous membranes lining the oral cavity were moderately hyperemic and normoperfused, normochromic, and normotonic.

### 2.2. Clinical Findings

The initial differential diagnosis was formulated based on clinical examination, taking into account the lesion′s location and morphological characteristics. Given its median dorsal position and erythematous appearance, the lesion was initially considered compatible with median rhomboid glossitis. The initial suspicion of median rhomboid glossitis was based on the lesion′s median dorsal location and erythematous appearance, which are typical features of this condition. Although the lesion did not present classic symptoms such as burning sensation, a trial of topical antifungal therapy was considered appropriate as a first‐line conservative approach. The lack of complete resolution and the subsequent increase in lesion volume prompted reconsideration of the initial diagnosis.

At the first follow‐up visit, a slight reduction in lesion size was observed. Continuation of topical therapy for an additional 7 days was therefore recommended, together with scheduling a subsequent follow‐up appointment and a concomitant cytological examination. Cytology was negative for fungal elements and pathogenic bacteria.

The patient′s parents were advised to monitor the oral cavity daily and to report any increase in lesion size or the appearance of new mucosal alterations. At the next clinical evaluation, however, a progressive increase in volume was noted, along with a more evident vascular component. Considering these changes, the patient was referred to the Maxillofacial Surgery Unit at Fondazione IRCCS Cà Granda Ospedale Maggiore Policlinico of Milan for further assessment.

Given the lesion′s evolution and vascular appearance, a vascular etiology was considered. The differential diagnosis was therefore expanded to include vascular malformations, such as infantile hemangioma and both high‐ and low‐flow venous malformations. A traumatic origin was excluded based on a negative history for recent or previous local injury. Burkitt lymphoma, which may present as a rapidly enlarging lingual mass in pediatric patients, was also ruled out through clinical evaluation and laboratory investigations.

### 2.3. Diagnostic Assessment

A single MRI examination of the head and neck was performed, including T1‐weighted imaging (T1WI), T2‐weighted imaging (T2WI), T2 fat‐suppressed, and postcontrast T1‐weighted sequences with fat suppression. MRI was performed using a standard pediatric head and neck protocol on a 1.5 Tesla scanner; technical parameters were consistent with routine clinical imaging protocols.

The lesion appeared well circumscribed and superficial, measuring approximately 15 × 8 × 13 mm. It was iso‐ to hypointense on T1‐weighted images and markedly hyperintense on T2‐weighted sequences. Mild, homogeneous enhancement was observed following gadolinium administration. No flow voids, arterial feeders, or deep tissue infiltration were identified.

**Figure 2:**
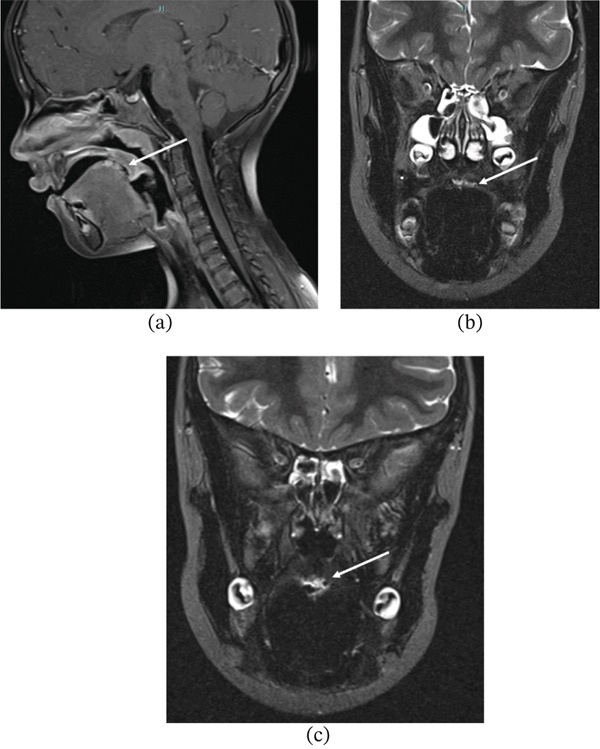
Magnetic resonance imaging of the lesion. (a) Sagittal T2‐weighted image showing a well‐defined hyperintense lesion (arrow) on the dorsal tongue. (b) Axial T2‐weighted image confirming superficial localization without deep infiltration (arrow). (c) Postcontrast T1‐weighted image with fat suppression demonstrating mild homogeneous enhancement (arrow). MRI findings were suggestive of a vascular lesion, possibly hemangioma, without definitive diagnostic features.

These findings were suggestive of a vascular lesion, possibly hemangioma, but were not diagnostic. (Figure 2a–c). At the follow‐up visit in April 2023, the overall appearance of the lesion was unchanged; there was a possible slight increase in thickness, but absence of related symptoms. Considering that the MRI findings were not conclusive, a surgical excisional biopsy was scheduled in May 2023. MRI sequences used in the evaluation of this lesion include T1WI, T2WI, and postcontrast T1WI. The lesion appeared hyperintense on T2‐weighted images and iso‐ to hypointense on T1‐weighted images, with mild contrast enhancement and no evidence of deep infiltration or flow voids. These findings were suggestive of a low‐flow vascular lesion. [18].

This case highlights the potential overlap of MRI features between hemangioma and lymphatic malformation, reinforcing the need for histological confirmation in atypical cases. Although ultrasound with Doppler is often recommended as a first‐line imaging modality in pediatric patients with suspected vascular lesions, MRI was preferred in this case to better evaluate lesion extent and its relationship with surrounding anatomical structures. In retrospect, Doppler ultrasound could have provided additional information regarding vascular flow characteristics and might have contributed to narrowing the differential diagnosis.

### 2.4. Therapeutic Intervention

The aim of the surgery was the excision of a known mucosal neoformation of the lingual dorsum and the histological diagnosis. The desired effect of surgical excision is to eliminate the lesion, preventing its recurrence, and to preserve the adjacent noble structures, ensuring the organ′s functionality over time and excellent healing.

The primary goal of treating lymphatic malformations is to restore or maintain functional and aesthetic integrity [19].

There is no standardized treatment in literature, and treatment is chosen according to the experience of the team handling the case, considering the individuality of the patient [20].

Treatment depends on its type, size, anatomical involvement, and invasion of surrounding tissue. Microcystic lesions are diffuse and difficult to eradicate, whereas macrocystic lesions are localized and can be easily eradicated [21].

During the hospitalization, the patient was in therapy with amoxicillin and clavulanic acid 300 mg three times a day and paracetamol. The rationale for perioperative antibiotic use in a pediatric patient is to reduce the risk of surgical site infection (SSI) due to the clean‐contaminated nature of oral cavity procedures and the potential for bacterial translocation during mucosal disruption.

**Figure 3:**
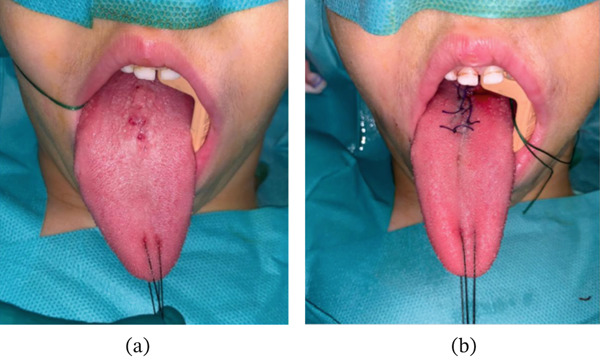
(a) Preoperative image of the lesion, (b) postoperative image with excision of the entire lesion.

Figure 3a shows the lesion before surgery. Under general anesthesia, excision of the known mucosal neoformation of the lingual dorsum was performed. After infiltration of local anesthetic and vasoconstrictor and positioning of a traction thread at the level of the lingual tip, the surgeon proceeds to exeresis of the pathological lingual mucosa in a diamond pattern using an electric scalpel. The hemostasis was controlled, and the piece was sent for histological analysis. The wounds were sutured with resorbable stitches (Figure 3b).

At the same time, surgical correction of the right indirect inguinal hernia was performed. The surgical correction of the right indirect inguinal hernia was a previously scheduled procedure and was performed during the same hospitalization for logistical reasons. It did not influence the diagnostic or therapeutic management of the oral lesion. At discharge, the patient was in good general condition, vital signs were normal, and the pain control was good.

Surgical wounds were in order; there were no active unpicked bleeds and no signs of infection. Edema and bruising were compatible with the date and method of intervention. The prognosis was 7 days, except for complications. The follow‐up visit was programmed 7 days after the surgery.

The home therapy was paracetamol according to weight as needed and antibiotic therapy for 7 days (amoxicillin and clavulanic acid 300 mg 3 times a day). The follow‐up instructions were as follows: avoid traumatism at the surgical site, warm and soft diet for 10 days, thorough oral hygiene immediately after meals with toothbrush, toothpaste, and mouthwash, avoid irritating the oral mucous membranes (spicy/acidic foods), keep the dressing in the right groin dry and clean until the follow‐up visit, avoid sports activity for 30 days, and avoid physical efforts.

### 2.5. Follow‐Up and Outcomes

The histopathological diagnosis evaluated a morphological and immunophenotypic finding compatible with macrocystic lymphatic malformation. The resection margins were lesion free. The immunohistochemical stains performed were podoplanin and Ki‐67, which were consistent. Ki‐67 immunohistochemical staining showed a very low proliferative index (estimated < 1%), supporting the nonneoplastic nature of the lesion.

**Figure 4:**
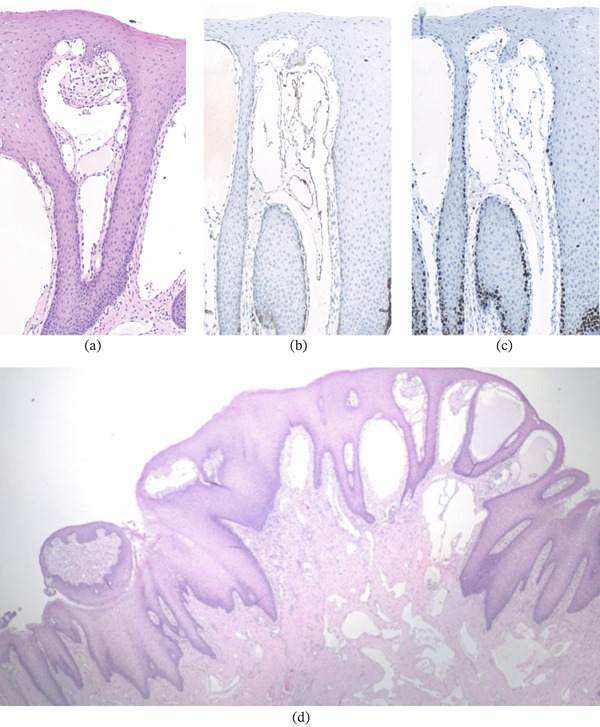
(a) Ki‐67 immunohistochemical staining showing a very low proliferative index (100× magnification). (b) Low‐power photomicrograph showing a domed‐shaped lesion with dilated vascular spaces within the chorion (hematoxylin–eosin, 40× magnification). (c) High‐power view demonstrating medium‐sized vascular spaces lined by bland endothelial cells (hematoxylin–eosin, 100× magnification). (d) Podoplanin immunohistochemical staining highlighting lymphatic endothelial cells (100× magnification).

There was no evidence of microorganisms by Periodic Acid–Schiff (PAS) and Grocott′s methenamine silver (GMS) staining (Figure 4).

**Figure 5:**
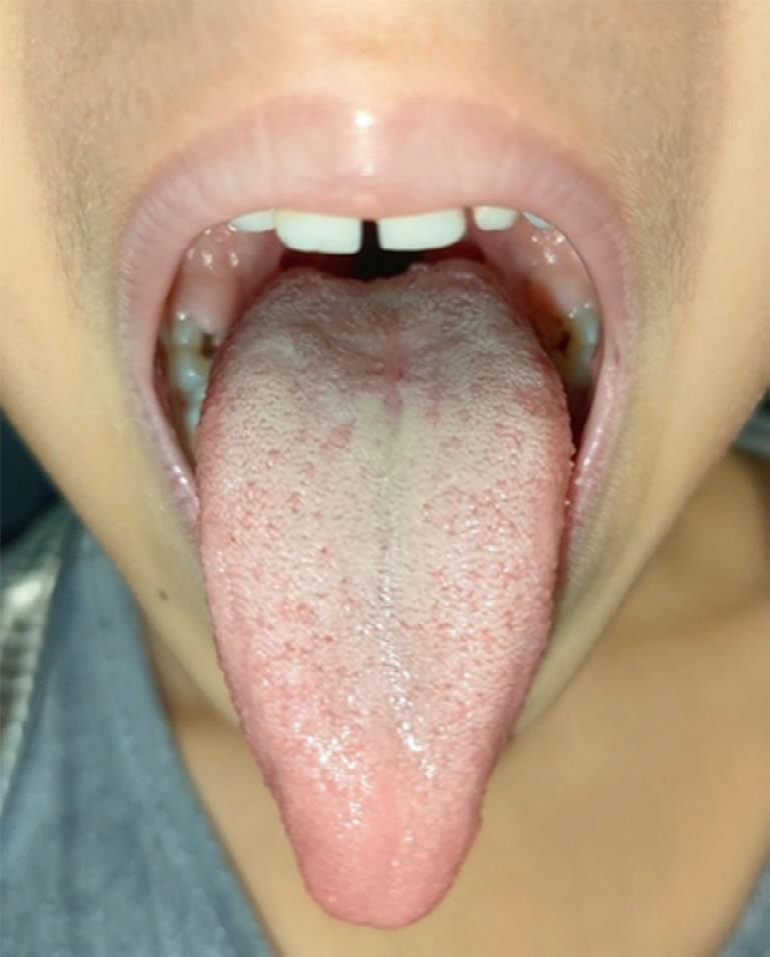
Wound healing 1‐month follow‐up.

At the follow‐up visit, performed 1 week after surgery, the surgical wound was healing, with central dehiscence. A soft and fresh diet was recommended in addition to accurate oral hygiene. One month after surgery the surgical site was completely healed, without complications, with total recovery of normal feeding (Figure 5). All postsurgical follow‐up visits were conducted at the Oral Medicine and Pathology Unit. Given the known risk of recurrence, particularly in lymphatic malformations of the tongue, a long‐term follow‐up program has been established. The patient is scheduled for periodic clinical evaluations at 6 and 12 months, with further follow‐up based on clinical findings.

### 2.6. Timeline of Clinical Events

Table 1 shows the summary of the patient′s clinical management, including initial presentation, diagnostic work‐up, therapeutic intervention, and follow‐up in accordance with CARE reporting guidelines.

**Table 1:** Summary of the patient′s clinical course, including initial presentation, diagnostic work‐up, therapeutic intervention, and follow‐up, in accordance with CARE reporting guidelines.

Timepoint	Clinical event
April 2022	Initial appearance of the lesion reported by the patient′s parents
December 2022	First clinical evaluation at the Oral Medicine and Pathology Unit
December 2022	Initial diagnosis of median rhomboid glossitis and start of topical antifungal therapy
January 2023	First follow‐up visit: partial reduction in lesion size
Early 2023	Cytological examination performed (negative for fungal elements and bacteria)
Subsequent follow‐up	Progressive increase in lesion volume and suspicion of vascular origin
April 2023	MRI of head and neck performed
May 2023	Surgical excision of the lesion and histopathological analysis
1‐week postoperative	Initial healing with partial dehiscence observed
1‐month postoperative	Complete healing with restoration of normal function
Planned follow‐up	Clinical evaluations scheduled at 6 and 12 months

## 3. Discussion

Lymphatic malformations of the tongue represent a clinically heterogeneous entity within the broader spectrum of vascular anomalies. According to the ISSVA classification, vascular malformations are structurally abnormal vessels without endothelial proliferation, distinguishing them from vascular tumors [22]. Lymphatic malformations are characterized by dilated lymphatic channels lined by endothelial cells and lacking encapsulation, features that account for their infiltrative behavior in certain anatomical regions [23].

The head and neck region is the most frequently affected anatomical site, accounting for approximately 75% of cases [24]. Within the oral cavity, the tongue—particularly its anterior portion—is the most common location, where lesions may present as localized nodules or diffuse microcystic involvement leading to macroglossia [25]. Diffuse microcystic lesions extending across the hyoid or involving multiple anatomical compartments are considered particularly challenging due to their infiltrative nature and higher functional impact [26].

The clinical differential diagnosis of a vascular‐appearing lingual lesion includes branchial and thyroglossal duct cysts [27], pyogenic granuloma [28], lipoma, mucocele, and hemangioma [29]. In small or superficially located lesions, clinical distinction may be difficult, especially when characteristic vesicular morphology is absent. In the present case, the lesion initially mimicked a benign mucosal condition, reflecting observations reported in the literature where early‐stage lymphatic malformations may present with nonspecific clinical features [30].

Diagnostic imaging plays a fundamental role in characterizing vascular anomalies. Ultrasound is often employed as a first‐line modality in pediatric patients because of its noninvasive nature and ability to identify cystic components [31–36]. However, MRI remains the most informative technique for evaluating lesion extent, internal composition, and relationships with adjacent anatomical structures [32, 34–37]. Typical MRI findings in lymphatic malformations include hyperintensity on T2‐weighted sequences and variable contrast enhancement consistent with low‐flow vascular characteristics. Nevertheless, radiologic overlap between lymphatic malformations and other low‐flow vascular lesions, including hemangiomas, has been documented [33, 38]. In the present case, MRI findings suggested a superficial low‐flow lesion but were insufficient to establish a definitive diagnosis, a limitation similarly described in previous studies [33, 38]. This diagnostic uncertainty justified histopathological confirmation.

Histological examination remains the diagnostic gold standard in equivocal cases [38]. Lymphatic malformations are defined by irregular, dilated lymphatic spaces lined by flattened endothelial cells with minimal cytologic atypia and low proliferative activity [32]. Immunohistochemical staining with podoplanin confirms lymphatic endothelial differentiation, whereas a low Ki‐67 index supports their nonneoplastic nature. The histopathological findings observed in our patient were consistent with these established criteria and excluded proliferative vascular tumors. An additional point of interest in this case is the apparent discrepancy between the clinical impression and the histopathological classification. Clinically, the lesion appeared small and superficially localized, features that may suggest a microcystic pattern. However, histological examination demonstrated a macrocystic lymphatic malformation. This discrepancy highlights the known limitations of clinical and radiological assessment in accurately defining the cystic subtype, particularly in small or early lesions. It is possible that the lesion contained cystic spaces not appreciable on imaging or clinical inspection. This finding further supports the role of histopathological evaluation as the definitive diagnostic tool in equivocal cases.

There are several options for treating lymphatic malformation, including surgery, sclerotherapy, cryotherapy, lasers, steroids, and bleomycin [39].

Surgical resection is the most common treatment, but high recurrence rates and potential complications such as bleeding, infection, and deformity have been observed [39–41]. Surgery is recommended more often than other treatment alternatives for lymphatic malformation of the tongue in pediatric patients because microcystic lymphatic malformations (the most common subtype affecting the tongue) are generally poorly responsive to sclerotherapy and other nonsurgical modalities. Surgical excision offers the highest likelihood of complete lesion control, especially when the lesion is well circumscribed and localized, and is often necessary to address functional impairments such as airway obstruction, feeding difficulties, speech problems, and recurrent infection or bleeding [31–33]. Sclerotherapy is effective primarily for macrocystic or mixed lymphatic malformations, but microcystic lesions—typical of lingual involvement—respond suboptimally, with lower rates of complete resolution and higher recurrence rates compared with surgery [31–33]. Minimally invasive approaches, such as laser photocoagulation, may be used for superficial lesions or as adjuncts, but are rarely curative for deeper or extensive disease [23–26]. Observation is reserved for minor, asymptomatic lesions, but is not appropriate for cases with significant functional compromise [36–38]. Surgical intervention, while associated with risks of complications and recurrence, remains the mainstay for symptomatic, microcystic, or refractory lymphatic malformation of the tongue in children, as supported by multiple retrospective series and consensus among pediatric otolaryngologists [38–40]. The decision is individualized based on lesion type, extent, and clinical impact.

Therapeutic strategies vary according to lesion morphology and anatomical extension [19, 21]. Macrocystic lesions tend to respond more favorably to sclerotherapy, including agents such as OK‐432, whereas microcystic lesions—more frequently observed in lingual involvement—are often less responsive to nonsurgical approaches and are associated with higher recurrence rates [41–43]. Laser‐based treatments, particularly CO_2_ laser therapy, have demonstrated favorable functional and hemostatic outcomes in selected superficial oral lesions [41, 44–46]. Bleomycin sclerotherapy has also shown efficacy in head and neck lymphatic malformations, with acceptable safety profiles in pediatric cohorts [43, 47].

More recently, pharmacologic therapies targeting the mTOR pathway have gained attention. Systemic sirolimus has been associated with reduction in lesion volume and improvement in quality of life in complex or refractory vascular anomalies [48–50], whereas topical sirolimus formulations have demonstrated promising preliminary results in localized microcystic lesions [51]. Additional pharmacologic approaches, including sildenafil therapy, have been explored in selected cases with reported symptomatic improvement [52]. Despite these advances, long‐term data remain limited, and treatment selection must be individualized.

In contrast to the diffuse or syndromic forms described in more complex series [48, 53], our patient presented with a localized lesion without airway compromise or significant functional limitation. Although lingual lymphatic malformations may impair swallowing, breathing, and dentition in extensive cases [53], the lesion in this case remained confined to the dorsal surface and did not involve deeper planes. Surgical excision was therefore considered an appropriate and definitive approach. Surgery remains a cornerstone of treatment for well‐circumscribed lesions or when diagnostic uncertainty persists [54–60]. Reported recurrence rates range from 10% to 53%, particularly in diffuse microcystic forms [54–60]; however, complete excision in localized lesions is associated with more favorable outcomes.

The favorable postoperative course observed in this patient—characterized by preservation of tongue mobility, restoration of feeding function, and absence of recurrence to date—supports existing evidence that early surgical management of circumscribed lesions can achieve durable functional resolution [54–60]. Moreover, the absence of airway involvement in this case highlights the spectrum of clinical severity reported in the literature and reinforces the importance of individualized risk assessment [61, 62].

Overall, this case aligns with previously published data emphasizing that imaging findings may be suggestive but not definitive, and that histopathological evaluation remains essential in diagnostically uncertain cases [45, 48]. It further illustrates that, despite the growing range of minimally invasive and pharmacologic options [52, 57–60, 63], surgical excision retains a central role in the management of selected pediatric lingual lymphatic malformations. Careful clinical evaluation, appropriate imaging, and multidisciplinary decision‐making remain fundamental to optimizing outcomes while preserving function and minimizing morbidity [61, 62].

## 4. Conclusion

Our case highlights that histological evaluation may be mandatory in suspected lymphatic malformations, given the nonspecific nature of clinical and radiological findings. Complete surgical excision not only confirmed the diagnosis but also resolved diagnostic uncertainty and demonstrated the effectiveness of surgery in the management of lingual lymphatic malformation. To date, no recurrence has been observed. Awareness of the possible occurrence of lymphatic malformations in uncommon sites such as the oral cavity is essential to prevent misdiagnosis, ensure timely initiation of appropriate therapy, and establish a correct follow‐up protocol, thereby avoiding unnecessary or ineffective treatments.

## Author Contributions

Conceptualization: F.D.M. and F.P.; methodology: F.D.M., M.P., and E.S.; software: M.P. and A.S.; validation: E.S.; formal analysis: F.P. and M.B.; investigation: F.D.M. and E.S.; resources: S.D. and F.P.; data curation: M.P.; writing—original draft preparation: S.D.; writing—review and editing: M.P. and A.G.; visualization: M.P. and E.K.; supervision: F.S. and A.S.; and project administration: F.S. and A.S.

## Funding

Open access publishing facilitated by Universita di Pavia, as part of the Wiley ‐ CRUI‐CARE agreement.

## Disclosure

All authors have read and agreed to the published version of the manuscript.

## Ethics Statement

This case report was exempt from ethics committee review as it describes routine clinical care without experimental intervention.

## Consent

Written approval and informed consent were obtained by the parents to publish case history and images.

## Conflicts of Interest

The authors declare no conflicts of interest.

## Data Availability Statement

The authors confirm that the data supporting the findings of this study are available within the article.

## Supporting information


**Supporting Information** Additional supporting information can be found online in the Supporting Information section. Table S1: CARE checklist.
