# Supplementary material for: Pediatric Lymphangioma of the Tongue, From Diagnosis to Surgery: A Case Report
Source: Case Rep Dent. 2026 May 3;2026:8570180. doi: 10.1155/crid/8570180 (PMC13136523; doi:10.1155/crid/8570180)
Supplement: Supplementary file 1 — Supporting Information Additional supporting information can be found online in the Supporting Information section. Table S1: CARE checklist. [file CRID-2026-8570180-s001.docx]

**Supplementary file:** Care checklist.


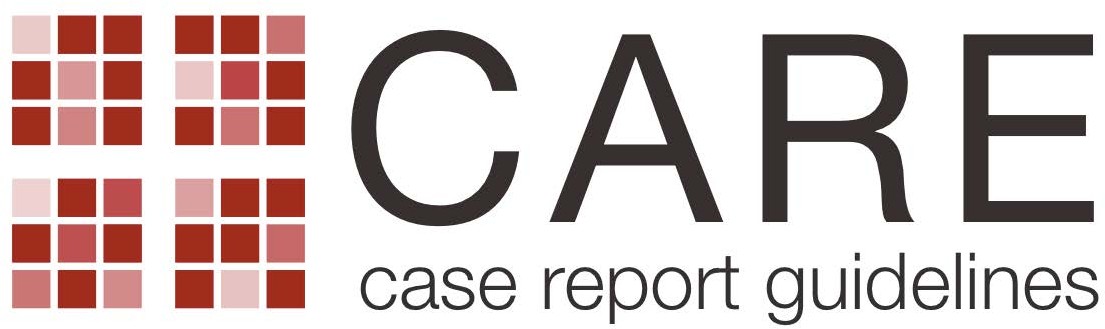
CARE Checklist of information to include when writing a case report
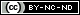


**Topic Item Checklist item description Reported on Line**

**Title 1** The diagnosis or intervention of primary focus followed by the words “case report” 1

**Key Words 2** 2 to 5 key words that identify diagnoses or interventions in this case report, including "case report" 47-48

**Abstract**

**(no references)**

**3a** Introduction: What is unique about this case and what does it add to the scientific literature? 52-55

**3b** Main symptoms and/or important clinical findings 46-48

**3c** The main diagnoses, therapeutic interventions, and outcomes 46-52

**3d** Conclusion—What is the main “take-away” lesson(s) from this case? 56-57

**Introduction 4** One or two paragraphs summarizing why this case is unique (**may include** reference**s**) 102-104

**Patient Information 5a** De-identified patient specific information 112-116

**5b** Primary concerns and symptoms of the patient 118-127

**5c** Medical, family, and psycho-social history including relevant genetic information 115-116

**5d** Relevant past interventions with outcomes N.R.

**Clinical Findings**

**Timeline**

**Diagnostic Assessment**

**Therapeutic Intervention**

**Follow-up and Outcomes**

1. Describe significant physical examination (PE) and important clinical findings 118-121
2. Historical and current information from this episode of care organized as a timeline N.R.

**8a** Diagnostic testing (such as PE, laboratory testing, imaging, surveys). 154-175

**8b** Diagnostic challenges (such as access to testing, financial, or cultural) 163-167

**8c** Diagnosis (including other diagnoses considered) 280-283

**8d** Prognosis (such as staging in oncology) where applicable N.R.

**9a** Types of therapeutic intervention (such as pharmacologic, surgical, preventive, self-care) 128-130,139-142,242-251

**9b** Administration of therapeutic intervention (such as dosage, strength, duration) 242-246

**9c** Changes in therapeutic intervention (with rationale) N.R.

**10a** Clinician and patient-assessed outcomes (if available) 268-276

**10b** Important follow-up diagnostic and other test results 280-301

**10c** Intervention adherence and tolerability (How was this assessed?) N.R.

**10d** Adverse and unanticipated events N.R.

**Discussion 11a** A scientific discussion of the strengths AND limitations associated with this case report 306-378

**11b** Discussion of the relevant medical literature **with references** 306-378

**11c** The scientific rationale for any conclusions (including assessment of possible causes) N.R.

**11d** The primary “take-away” lessons of this case report (without references) in a one paragraph conclusion 381-388

**Patient Perspective 12** The patient should share their perspective in one to two paragraphs on the treatment(s) they received N.R.

**Informed Consent 13** Did the patient give informed consent? Please provide if requested . . . . . . . . . . . . . . . . . . . . . . . . . . . . . . . . . . . . . . **Yes No**
